# The Amyloid Precursor Protein is rapidly transported from the Golgi apparatus to the lysosome and where it is processed into beta-amyloid

**DOI:** 10.1186/s13041-014-0054-1

**Published:** 2014-08-01

**Authors:** Joshua HK Tam, Claudia Seah, Stephen H Pasternak

**Affiliations:** 1 J. Allyn Taylor Centre for Cell Biology, Molecular Brain Research Group, Robarts Research Institute, 100 Perth Drive, London N6A 5K8, Ontario, Canada; 2 Department of Clinical Neurological Sciences, London N6A 5K8, Ontario, Canada; 3 Department of Physiology and Pharmacology, Schulich School of Medicine and Dentistry, the University of Western Ontario, London N6A 5K8, Ontario, Canada

**Keywords:** Lysosome, Live cell imaging, Confocal microscopy, Amyloid Precursor Protein, Trafficking, Beta amyloid production, Gamma-secretase

## Abstract

**Background:**

Alzheimer’s disease (AD) is characterized by cerebral deposition of β-amyloid peptide (Aβ). Aβ is produced by sequential cleavage of the Amyloid Precursor Protein (APP) by β- and γ-secretases. Many studies have demonstrated that the internalization of APP from the cell surface can regulate Aβ production, although the exact organelle in which Aβ is produced remains contentious. A number of recent studies suggest that intracellular trafficking also plays a role in regulating Aβ production, but these pathways are relatively under-studied. The goal of this study was to elucidate the intracellular trafficking of APP, and to examine the site of intracellular APP processing.

**Results:**

We have tagged APP on its C-terminal cytoplasmic tail with photoactivatable Green Fluorescent Protein (paGFP). By photoactivating APP-paGFP in the Golgi, using the Golgi marker Galactosyltranferase fused to Cyan Fluorescent Protein (GalT-CFP) as a target, we are able to follow a population of nascent APP molecules from the Golgi to downstream compartments identified with compartment markers tagged with red fluorescent protein (mRFP or mCherry); including rab5 (early endosomes) rab9 (late endosomes) and LAMP1 (lysosomes). Because γ-cleavage of APP releases the cytoplasmic tail of APP including the photoactivated GFP, resulting in loss of fluorescence, we are able to visualize the cleavage of APP in these compartments. Using APP-paGFP, we show that APP is rapidly trafficked from the Golgi apparatus to the lysosome; where it is rapidly cleared. Chloroquine and the highly selective γ-secretase inhibitor, L685, 458, cause the accumulation of APP in lysosomes implying that APP is being cleaved by secretases in the lysosome. The Swedish mutation dramatically increases the rate of lysosomal APP processing, which is also inhibited by chloroquine and L685, 458. By knocking down adaptor protein 3 (AP-3; a heterotetrameric protein complex required for trafficking many proteins to the lysosome) using siRNA, we are able to reduce this lysosomal transport. Blocking lysosomal transport of APP reduces Aβ production by more than a third.

**Conclusion:**

These data suggests that AP-3 mediates rapid delivery of APP to lysosomes, and that the lysosome is a likely site of Aβ production.

## Introduction

AD is the leading cause of dementia in adults [1]. A neuropathological hallmark of AD is the accumulation of β-amyloid (Aβ) in plaques in the brain [2]. Aβ is produced through sequential cleavage of amyloid precursor protein (APP) by secretases. Cleavage by β-secretase removes the N-terminal ectodomain, leaving a 99 residue C-terminal fragment (CTF) containing Aβ [3]–[5]. The CTF is then processed by γ-secretase [6] to produce Aβ species ranging from 39–43 residues in length [7]. The 42 amino acid form of Aβ (Aβ42) has a higher propensity to aggregate, is more toxic in cell culture experiments and is the dominant component of amyloid plaques [8]–[11].

Many experiments suggest that the production of Aβ occurs in the endosomal/lysosomal system. Work in our laboratory has demonstrated that lysosomes are highly enriched in APP and γ-secretase proteins (composed of at least presenilin, APH1, PEN-2 and nicastrin) and γ-secretase activity (the ability to cleave APP to produce Aβ) [12],[13]. Others have also described APP and γ − secretase activity in lysosome-related autophagosomes and phagosomes [14],[15]. In agreement with these findings, deacidification of the endosomal/lysosomal system decreases Aβ production [16],[17]. When proteolysis is blocked with protease inhibitors or by presenilin knock-out (which abolishes γ-secretase activity), amyloidogenic fragments of APP accumulate in lysosomes [18]–[20].

While many studies have shown that endocytosis of APP is crucial for Aβ production [21]–[23], a number of studies have suggested that the intracellular trafficking of APP might also play an important role in Aβ generation. For example, Aβ production is decreased in MDCK cells when APP is sorted to the basolateral membrane [24]. More recent studies demonstrate that Aβ production is decreased by retrograde sorting of APP from endosomes to the trans-Golgi network (TGN) [25]–[28]. Because the TGN serves as sorting station for nascent cargo from the ER and protein recycled from endosomes [29], an understanding the trafficking of APP into and out of the Golgi will increase our understanding of Aβ production.

While cell-surface proteins are amendable to many labeling techniques, intracellular proteins are more difficult to track. Photoactivatable-Green Fluorescent Protein (paGFP) has provided a new tool to examine intracellular trafficking. paGFP is a form of GFP that has low basal fluorescence, but develops strong, stable green fluorescence after being activated by 413 nm laser light [30],[31]. Constructs using paGFP [30],[32] have been used to examine the turnover of peroxisomes [33] and autophagosomes [34] and to examine actin dynamics in neuronal dendritic spines [35].

Fluorescent protein tags have previously been used to examine movement of APP containing vesicles [36],[37], including tubular-vesicular structures emanating from the Golgi apparatus [38]. APP-paGFP constructs have been used to visualize APP undergoing fast axonal transport [39] and to image the trafficking of APP out of the perinuclear region, although these authors did not identify the compartments involved [28],[40].

Our aim was to examine the trafficking of APP from the Golgi apparatus and to identify downstream compartments and identify sites of cleavage. We used targeted activation of APP-paGFP in the Golgi apparatus (identified using Galactosyltransferase fused to Cyan Fluorescent Protein (GalT-CFP) [41], and followed activated APP-paGFP using confocal microscopy fluorescence imaging to intracellular compartments labeled with compartment marker proteins fused to red fluorescent proteins including rab5 (early endosomes) [42]–[44], rab9 (late endosomes) [45],[46] and LAMP1 (lysosomes)[47],[48]. Tracking the disappearance of green fluorescent APP-paGFP from these downstream compartments allows us to examine the intracellular site of cleavage and degradation; essentially performing pulse chase experiments in single cells. Surprisingly, we show that a large fraction of APP traffics rapidly to LAMP1-labeled lysosomes within seconds after photoactivation in the Golgi, and is subsequently cleaved by a γ-secretase-like activity. This pathway is mediated by an interaction between APP and Adaptor Protein 3 (AP-3). Knocking down AP-3 blocks lysosomal transport and reduces Aβ secretion into the media for more than one third. This suggests that direct lysosomal transport of APP is an important source of Aβ.

## Results

### APP-paGFP can be followed as it traffics from the Golgi apparatus to LAMP1-labeled compartments

**Figure 1:**
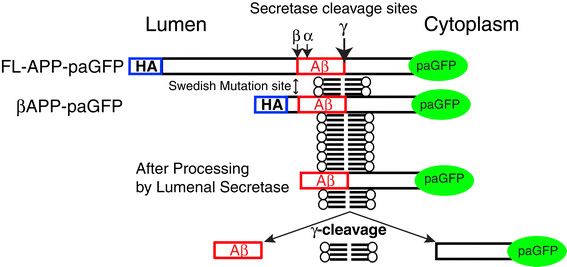
**Overview of constructs.** For these experiments, APP constructs were generated including the full length APP 751 fused to paGFP on its C-terminus. A shorter construct consisting of the C-terminal 112 amino acids of APP fused to paGFP. Both constructs include a linker with includes an N-terminal HA epitope tag, and both constructs contain α-, β- and γ- cleavage sites. Cleavage at the γ-site will release the C-terminal tail of APP along with the paGFP tag into the cytoplasm.

In order to study the intracellular trafficking of APP from the Golgi apparatus in live cells, we generated expression constructs (Figure 1) containing full length APP (FL-APP) fused to an N-terminal HA epitope tag and photoactivatable Green Fluorescent Protein (paGFP) at its C-terminal cytoplasmic tail. To avoid any confounding effects of uncharacterized N-terminal APP cleavage and sorting signals [49] we also examined a shortened construct (referred to as βAPP) fused to the C-terminal 112 amino acids of APP containing both the β- and γ-cleavage sites. This construct also contains an N-terminal HA- epitope tag. βAPP-CFP colocalizes with full length FL-APP-GFP and has the same subcellular distribution as endogenous APP in primary neurons [50]. These constructs are cleaved by secretases (Additional file 1: Figure S1) and produce Aβ (ELISA data below). In fixed cells, the N-terminal HA-tag of these constructs are well colocalized with the C-terminal Fluorescent protein tag, implying that much of the intracellular APP is trafficked before cleavage (Additional file 2: Figure S2). We have previously demonstrated that βAPP-CFP and FL-APP-GFP are also trafficked to the cell surface and internalized to endosomes and lysosomes [50]. After photoactivation of βAPP-paGFP and FL-APP-paGFP constructs, regions or compartments exhibiting APP accumulation of Golgi-derived APP will therefore appear as regions of increased green fluorescence. At sites where γ-cleavage occurs, cleavage will release the APP C-terminal and its paGFP tag into the cytoplasm, decreasing the fluorescent signal over time.

These studies were performed in the SN56 cell line, an easily transfectable cholinergic cell line [51],[52]. For each experiment, SN56 cells were co-transfected with an APP-paGFP construct along with a marker of the Golgi apparatus (GalT-CFP) [41] and subcellular compartment marker fused to a red fluorescent protein (mRFP or mCherry). Compartment markers were LAMP1 (lysosomes), Rab5 (early endosomes) and Rab9 (late endosomes). LAMP1 is a 120kDa protein that is localized to the limiting membrane of lysosomes [47],[48]. Rab5 localizes to the early endosomal membrane and is involved in homoegenous or heterogenous vesicle fusion [42]–[44]. Rab9 localizes to late endosomal membrane and evidence suggests that Rab9 is involved in returning cargo from the late endosome to the Golgi [45],[46]. Although many authors use rab7 as a late endosomal marker, rab7 also labels lysosomes extensively [53]. Cells with normal morphology, no inclusions, and normal distribution of compartment marker expression were imaged live on a Zeiss LSM510 laser scanning confocal microscope. Regions of interests (ROIs; the irradiation targets, typically 0.1-0.2 μm^2^) were drawn on the Golgi apparatus using the Zeiss Physiology package. During a 15-minute photoactivation period, cells were alternately imaged and then briefly irradiated with 405 nm laser light (25 mW) for 20 iterations (typically 2 seconds) within each of the ROI’s to photoactivate APP-paGFP to produce a video time course. The irradiation targets were carefully monitored throughout the experiments to ensure that they did not drift outside the Golgi apparatus. Because of the very small irradiation targets, and APP’s rapid movement through the Golgi apparatus, multiple rounds of photoactivation were required to create a strong green fluorescent signal. Images were acquired after each photoactivation cycle, approximately every 30 seconds, and colocalization analysis was performed using Imaris software (Bitplane). After the initial photoactivation period, cells were imaged for up to an hour to follow the movement of APP out of the Golgi and its clearance.

When we started these experiments, we expected that APP to move primarily to the cell surface and then to be internalized into lysosomes after 30 minutes to 1 hour. Instead, within seconds of photoactivation, activated bright green fluorescent APP-paGFP colocalized with LAMP1-mRFP, implying rapid transport to lysosomes. A typical experiment is shown in Additional file 3: Video S1, where βAPP-paGFP from the Golgi apparatus (blue) and can be seen moving within seconds to lysosomes (red). After 15 minutes of alternately photoactivating and imaging, cells were imaged (chased) for a further hour. During the chase period most of the green fluorescent APP disappeared, suggesting that it was being cleared.

**Figure 2:**
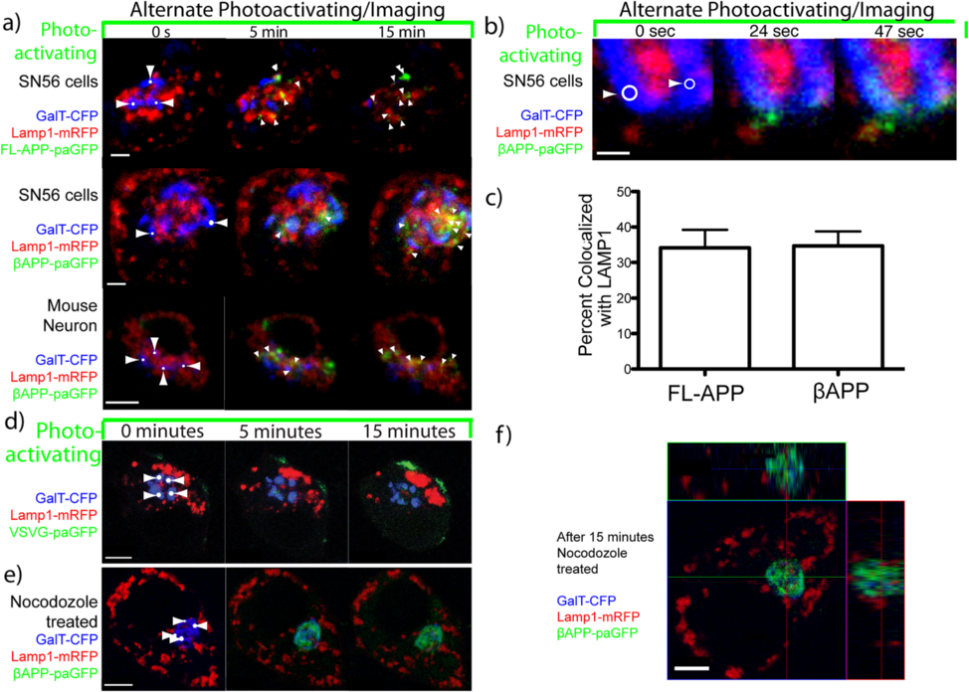
**APP is rapidly trafficked from the Golgi apparatus to LAMP1-labeled compartment.** Photoactivation targets were drawn on the Golgi apparatus (white dots with arrows). For 15 minutes, cells were alternately imaged and irradiated (photoactivated) with 405 nm laser light within the targets. White arrowheads point to APP-paGFP colocalized with Lamp1-mRFP. Scale Bar = 5 μm. **a)** Demonstrates rapid transport of Full length-APP- paGFP (top panel) and βAPP-paGFP (middle panel) from Golgi apparatus to a LAMP1-labeled compartment. The same trafficking occurs in mouse primary neurons (lower panel). (See Additional file 3: Video S1). **b)** Higher magnification images of βAPP-paGFP trafficking rapidly to a LAMP1 labeled compartment. Scale Bar = 1 μm. **c)** Comparison of the colocalization of activated FL-APP-paGFP (n = 9) and activated βAPP-paGFP (n = 8). **d)** SN56 cells transiently transfected with the secretory protein Vesicular Stomatitis Virus Glycoprotein-paGFP (VSVG-paGFP), GalT-CFP (blue), and LAMP1-mRFP (red) to demonstrate that very little of the green photoactivated VSVG-paGFP arrives in the LAMP1 compartment; paGFP does not alter trafficking. Scale bars = 5 μm. **e)** Transfected SN56 cells were treated for 5 minutes with nocodazole before imaging (See Additional file 5: Video S2) Scale bars = 5 μm. **f)** Z-stack of the same cell taken immediately following 15 minutes of photoactivation demonstrating that green signal remains inside the Golgi.

In these experiments, photoactivated both FL-APP-paGFP and βAPP-paGFP appear to be rapidly colocalized with LAMP-1 compartment (Figure 2a; top and middle panels). To confirm that this trafficking occurs in neurons, we then transfected GalT-CFP, βAPP-paGFP and LAMP1-mRFP into primary mouse cortical neurons. After photoactivating βAPP-paGFP in the Golgi, green fluorescence appears within 30 seconds to a minute in LAMP1-mRFP labeled compartments. (Figure 2a; bottom panel). To further demonstrate that the LAMP1 compartment rapidly received photoactivated APP-paGFP, we performed imaging at high magnification in closely cropped cells with βAPP-paGFP. In the earliest time points, it was possible to observe green fluorescent APP-paGFP arriving rapidly within LAMP1 compartments (Figure 2b). We quantitated the fraction of fluorescent activated paGFP colocalized with LAMP-mRFP after 15 minutes of photoactivation, we found that 34.14 ± 5.10% (Mean ± SEM) of FL-APP and 34.70 ± 4.05% of βAPP was colocalized with LAMP1 (not statistically different) (Figure 2c). Because the trafficking of the shorter construct was indistinguishable and resulted in brighter images, the βAPP-paGFP construct was used for the remainder of these experiments. Enlarged images from these experiments along with colocalization analysis is shown in Additional file 4: Figure S3. These images are very similar to the trafficking of LAMP1-paGFP from the Golgi to lysosomes produced by Lippincott-Schwartz [30].

To rule out the possibility that transport to LAMP1 compartment was due to overexpression or the paGFP tag, we examined the transport of the Vesicular Stomatitis Virus Glycoprotein (VSVG); a classic secretory trafficking protein. VSVG was tagged with paGFP at its cytoplasmic C-terminal [54],[55] similarly to our APP constructs. We transfected SN56 cells VSVG-paGFP. These cells were subjected to the same imaging protocol as βAPP-paGFP transfected cells. After the 15-minute pulse-period, VSVG-paGFP appeared on the cell surface and exhibited minimal transport to a LAMP1 compartment (Figure 2d), in concordance with previous results [32],[55]. Therefore, we conclude the paGFP tag did not alter the trafficking of APP.

To verify the accuracy of βAPP-paGFP photoactivation in the Golgi apparatus and that we were not photoactivating βAPP-paGFP in nearby structures, SN56 cells were transfected with GalT-CFP, βAPP-paGFP and LAMP1-mRFP and pretreated with nocodazole and/or cytocholasin D to block transport out of the Golgi [56]. Cells were then photoactivated and imaged for 15 minutes using the Golgi apparatus marker GalT-CFP as a target, and a Z-stack was taken immediately after the photoactivation period. During the entire experiment, photoactivated APP-paGFP remained almost exclusively within the Golgi apparatus (Figure 2e; Additional file 5: Video S2). Inspection of the post-irradiation Z-stack also revealed that photoactivated βAPP-paGFP was localized principally within the Golgi apparatus, with almost no fluorescence evident in other compartments or at the cell surface (Figure 2f).

### APP-paGFP traffics preferentially to lysosomes from the Golgi apparatus

Next, we examined APP trafficking from the Golgi apparatus to early and late endosomes. In these experiments, βAPP-paGFP was co-transfected with GalT-CFP along with either rab5- mRFP (early endosomes) or rab9-mCherry (late endosomes). Rab5 is highly associated with early endosomal membranes and is routinely used as a marker for early endosomes [42],[43]. Rab9 localizes to late endosomal membrane and evidence suggests that Rab9 is involved in returning cargo from the late endosome to the Golgi [45],[46],[53]. Rab7 is also a late endosomal marker [45], but Rab7 also defines a population of lysosomes [53]. Therefore, to avoid confounding late endosomes with lysosomes we chose Rab9 as our late endosomal marker.

**Figure 3:**
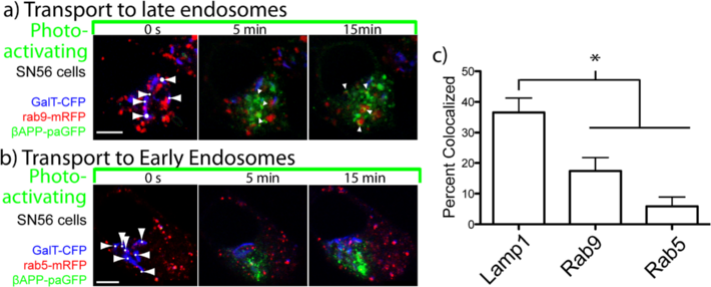
**APP is primarily transported to a LAMP1 compartment.** SN56 cells were cotransfected with plasmids expressing APP-paGFP, GalT-CFP (blue), and a compartment marker (red). Photoactivation targets were drawn on the Golgi apparatus (white dots with arrows). βAPP-paGFP trafficking was visualized from the from Golgi apparatus to Rab 9 labelled late endosomes **(a)** and Rab 5 labelled early endosomes **(b)**. Scale bars represent 5 μm. **c)** Percent of APP-paGFP fluorescence colocalized with respective compartment markers after 15 minutes of photoactivation in the Golgi (circles: LAMP1 (n = 9), squares: Rab9 (n = 10), triangles: Rab5 (n = 7)). Error bars represent standard deviation. (* = p < 0.05).

Cells were then alternately irradiated with 405 nm within targets placed over the Golgi apparatus and imaged to produce a time course of images. In these experiments, a small amount of βAPP-paGFP can be seen colocalizing with Rab9 and Rab5 (Figure 3a and b; respectively) at the end of the photoactivation period. At the end of the 15-minute pulse period, 36.57 ± 4.69% (Mean ± SEM) of photoactivated βAPP-paGFP colocalized with LAMP1 labeled compartments. Trafficking to rab9 and rab5 compartments was significantly lower at 17.39 ± 4.37% and 5.84 ± 3.05% respectively (Figure 3c). Although some LAMP1 labeling is found in early and late endosomes, the fact that significantly more APP was co-localized with LAMP1 than rab5 or rab9 suggests that APP is in bona fide lysosomes.

### APP-paGFP is cleaved in a LAMP1 positive compartment

**Figure 4:**
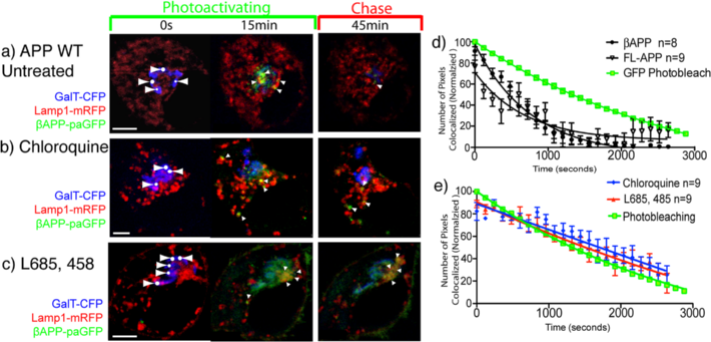
**APP is processed in the lysosome by a γ-secretase like activity.** SN56 cells were transiently transfected with βAPP-paGFP, GalT-CFP, and LAMP1-mRFP. Cells were alternately photoactivated with 405 nm light and imaged in the Golgi for 15 minutes, and then imaged every 30 seconds for 1 hour. **a)** Shows the accumulation of photoactivated APP-paGFP in the lysosome after 15 minutes, follow by its near complete clearance after 45 minutes. Arrowheads denote areas of colocalization. (See also Additional file 3: Video S1) **b)** Transiently transfected SN56 cells were pretreated with 100 μM chloroquine for 30 minutes prior to imaging. After chloroquine treatment APP is still visible in lysosomes after 45 minutes (See Additional file 6: Video S3). **c)** Cells treated with 0.5 μM L685, 458 (γ-secretase inhibitor) overnight prior to photoactivating/imaging. L685, 458 treatment substantially increases the accumulation of photoactivated βAPP-paGFP in lysosomes, and substantially decreases its cleance. Scale bars represent 5 μm (See Additional file 7: Video S4). **d)** Cleavage of βAPP-paGFP was determined by measuring the loss of FL-APP (black open triangles) and βAPP-paGFP (black closed circles) from LAMP1 labeled compartments. Values were averaged and normalized to begin at 100%. Overlaid in green squares is the loss of fluorescence of EGFP in the identical imaging protocol. Error bars represent SEM. (* = p < 0.05) **(e)** Shows the clearance of photoactivated APP-paGFP cells that were treated with 100 μM chloroquine for 30 minutes before imaging (n = 9) or with 0.5 μM L685, 458 (γ-secretase inhibior) (n = 9). Error bars represent SEM.

Subcellular fractionation has shown that γ-secretase proteins and APP are *bona fide* residents of the lysosomal membrane [13]. Furthermore, *in vitro* assays revealed that γ-secretase has an acidic optimal pH (4.5-5) [13]. Therefore, we hypothesized that inhibiting γ-secretase or lysosomal enzyme function could inhibit both secretase cleavage and nonspecific degradation of βAPP, which would result in paGFP fluorescence accumulation at the lysosome membrane. Therefore, we followed the extinction of paGFP fluorescence from LAMP1 positive vesicles after the end of the photoactivation period. We found that there was nearly complete extinction of photoactivated βAPP-paGFP (Figure 4a; Additional file 3: Video S1) and FL-APP-paGFP (not shown) fluorescence from the LAMP1 compartment within 1 hour. First we examined the effects of nonspecific inhibitor of lysosomal function using chloroquine. Chloroquine has been reported to alkalinized the endosomal/ lysosomal system and to inhibits APP clearance and Aβ production [17],[57],[58]. Cells were acutely treated with 100 μM chloroquine for 30 minutes before imaging. The increase in pH was confirmed by loss of LysoSensor Green signal (a pH dependent fluorescent probe, Invitrogen) from LAMP1 compartments (data not shown). As in the untreated control cells, βAPP-paGFP fluorescent signal was observed trafficking directly from the Golgi apparatus to LAMP1-mRFP labeled lysosomes (Figure 4b, Additional file 6: Video S3). However, cells treated with chloroquine accumulated fluorescent green photoactivated βAPP-paGFP in LAMP1 labeled compartments.

Next we assessed the ability of the highly potent and specific γ-secretase inhibitor L685,458 [59] to block the cleavage of βAPP-paGFP. SN56 cells were pretreated with 0.5 μM of L685,458 for 24 hours before imaging. L685,458 treatment caused marked accumulation photoactivated βAPP-paGFP in lysosomes during the photoactivation phase, and significantly reduced the clearance of APP from lysosomes. (Figure 4c; Additional file 7: Video S4).

We hypothesized that if APP were cleaved at the lysosomal membrane by secretase enzymes, the cytoplasmic tail of APP along with activated paGFP would be released into the cytoplasm resulting in loss of fluorescence from this compartment. Furthermore, this process would appear with first order kinetics. To quantitate APP-paGFP clearance from the lysosome after the photoactivation period, we measured the number of pixels of APP-paGFP fluorescence colocalized with LAMP1-mRFP using Imaris software for each time point, normalizing the highest value of colocalization (after the photoactivation period) to 100%. In these experiments, FL-APP-paGFP and βAPP-paGFP disappear from the lysosomes with non-linear kinetics. The disappearance of βAPP-paGFP was modeled using Prism 5 (GraphPad, La Jolla, CA) from the lysosome can be accurately modeled using the integrated rate equation for a first order reaction (k = 0.00153, r^2^ = 0.96) (Figure 4d). This suggests that APP is cleaved enzymatically in lysosomal compartments.

Because of our long imaging protocol, some of the loss fluorescence from activated paGFP could be the result of photobleaching. Therefore, we constructed photobleaching curves using enhanced-GFP (EGFP), as EGFP and paGFP have nearly identical photobleaching characteristics [30]. SN56 cells were transiently transfected with EGFP, and fixed with 4% paraformaldehyde. Cells were then imaged using the same imaging protocol, as in the previous live cell imaging experiments. The normalized number of green pixels at each time point was quantitated, and plotted on the same graph as our βAPP-paGFP clearance data (Figure 4d). βAPP-paGFP fluorescence decayed faster and became significantly lower than GFP fluorescence (One-way ANOVA; Tukey’s Post Hoc; p < 0.05). Therefore, the loss of βAPP-paGFP fluorescence appears to be the result of a first order enzymatic reaction (Figure 4d).

Next, we quantitated the effect of inhibitors on APP clearance (Figure 4e). After chloroquine treatment, photoactivated APP-paGFP in the lysosome decreased linearly over time during the chase phase, suggesting that it was not cleared by an enzymatic cleavage. There was no significant difference from loss of fluorescence due to photobleaching (One-way ANOVA; Tukey’s Post Hoc p < 0.05) (Figure 4e). In cells treated with L685,458 or chloroquine, the loss of βAPP-paGFP fluorescence from lysosomes during the chase phase was also not significantly different from the rate of loss of GFP fluorescence due to photobleaching (One-way ANOVA; Tukey’s Post Hoc; p < 0.05) (Figure 4e). Cells treated with L685,458 also consistently exhibited accumulation of photoactivated βAPP-paGFP at the cell surface (Figure 4c middle and right panels). This is in agreement with a previous study that showed APP internalization was decreased by treatment with γ-secretase inhibitors [60],[61]. Our data therefore suggests that βAPP-paGFP clearance from lysosomes is perfomed by both a pH-dependent protease (as expected for a β-secretase) and by the γ-secretase. The accumulation of APP in these LAMP1 compartments following inhibition of proteases suggests that they represent terminal lysosomes and not an endosomal intermediate.

### The Swedish mutation dramatically increases APP clearance from the lysosome, but not the Golgi apparatus

**Figure 5:**
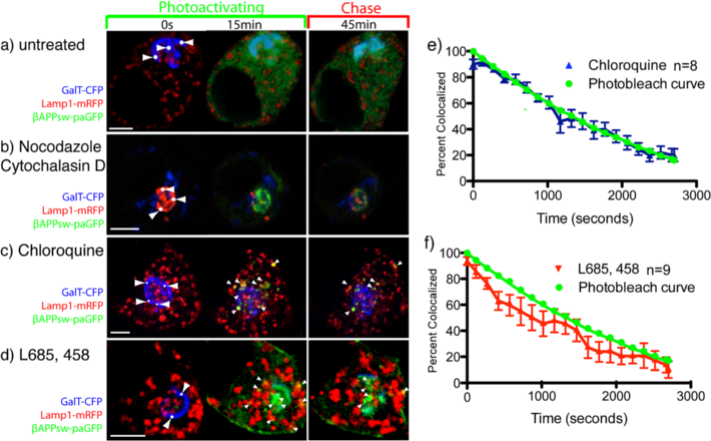
**The Swedish mutation causes rapid clearance of APP from lysosomes.** SN56 cells were transiently transfected with βAPPsw-paGFP, GalT-CFP, and LAMP1-mRFP. Scale bars represent 5 μm. **a)** βAPPsw-paGFP was photoactivated for 15 minutes in the GalT-CFP labeled compartment, and chased for 45 minutes. βAPPsw is cleaved nearly instantaneously and appears in the cytoplasm. **b)** Cells were treated for 5 minutes before imaging with 66 μM nocodazole and 10 μM cytochalasin. GalT-CFP is false colored red to provide better contrast, and LAMP1-mRFP has been false coloured blue. Photoactivated βAPPsw-paGFP accumulates in the Golgi and does not appear to be cleaved. **c)** Cells were treated acutely with 100 μM chloroquine (See Additional file 10: Video S7) which results in photoactivated βAPPsw-paGFP accumulating in lysosomes. White arrowheads represent βAPPsw-paGFP colocalized with LAMP1-mRFP **d)** Cells were treated with 0.5 μM L658, 458 (See Additional file 11: Video S8), which also causes photoactivated βAPPsw-paGFP to appear in lysosomes. Scale bars represent 5 μm. Quantitation of colocalized green pixels with LAMP1-mRFP show that the clearance of βAPPsw-paGFP from the lysosome proceeds linearly after treatment with **e)** chloroquine (n = 8), or with **f)** L658, 458 (n = 9). Error bars represent standard deviation.

The Swedish mutation (APPsw) is a double mutation at codons APP 670/671 (numbered in APP695) adjacent to the β-secreatse cleavage site that increases the rate of β-cleavage of APP by up to a factor of 10, and has been suggested to alter the trafficking of APP [22],[62],[63]. To examine effect of the Swedish mutation on intracellular APP trafficking, we transiently transfected SN56 cells with βAPPsw-paGFP along with plasmids expressing compartment markers for the Golgi apparatus and lysosomes. The same pulse-chase paradigm was performed on these cells, targeting the GalT-CFP labeled Golgi apparatus to photoactivate βAPPsw-paGFP. Unlike cells transfected with the wild type construct, green fluorescence did not accumulate in cells transfected with βAPPsw-paGFP in a LAMP1-labeled compartment. Instead, these cells rapidly developed diffuse green fluorescence throughout the entire cell body (Figure 5a; Additional file 8: Video S5). The diffuse cytoplasmic appearance of paGFP fluorescence suggests that APP is being rapidly cleaved, with the APP C–terminal fused to paGFP diffusing rapidly into the cytosol. This likely reflects the higher rate of β-cleavage of Swedish mutation [64],[65].

It has previously been suggested that secretase cleavage of the APPsw might occur in the Golgi apparatus. In order to examine APP cleavage in the Golgi apparatus, we repeated this experiment after treating the cells with nocodazole and cytocholasin D (Figure 5b; Additional file 9: Video S6). In dramatic contrast to untreated cells, virtually all of the paGFP fluorescence remains localized to the Golgi apparatus during the photoactivation period. Cells were then followed out to 1-hour post photoactivation, during which most of the green signal remains in the Golgi apparatus. It was not possible to quantify the clearance of APP from the Golgi apparatus in this experiment, because of photobleaching of the GalT-CFP marker. Although it is not possible to say from this data that there is no cleavage of APP in the Golgi apparatus, the Golgi apparatus does not appear to facilitate the majority of APP processing.

**Figure 6:**
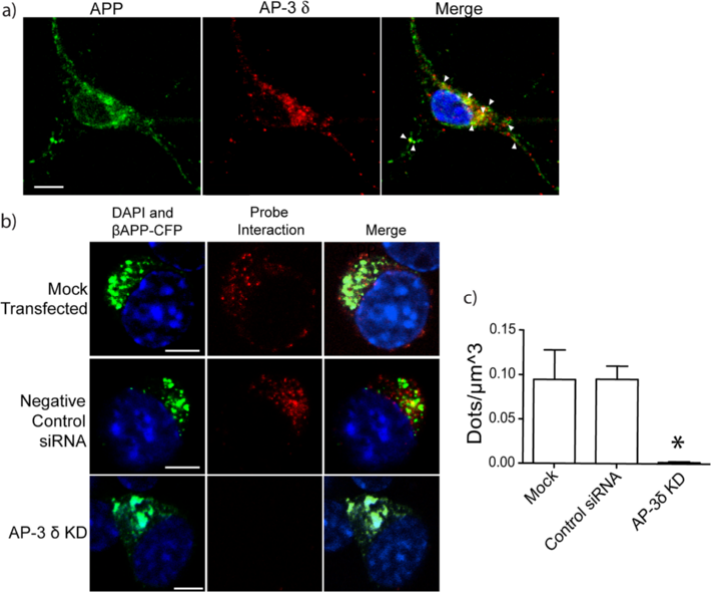
**AP-3δ and APP colocalize and interact. a)** E15 mouse neurons were cultured and immunostained with antibodies against AP-3δ (SA4; red) and APP (APP C-terminal; green). Arrowheads point to colocalized pixels. Scale bars represent 5 μm. Inset shows magnified view of the cell body. **b)** Proximity ligation assay (PLA) demonstrates the interaction of APP and AP-3δ. Cells were transiently transfected with βAPP-CFP with no siRNA, control siRNA or AP-3δ siRNA. Cells were stained with mouse anti- AP-3δ and rabbit anti-APP C-terminal antibodies. These were detected with secondary antibodies conjugated to complementary DNA sequences. When proteins are within 40 nm, DNA is ligated and replicated and detected by *in-situ* fluorescent red dots. AP-3δ siRNA substantially reduces the number of red dots. (scale bars represent 10 μm) **c)** Quantification of PLA fluorescent dots in SN56 cells normalized to cell volume (*p < 0.05).

In order to examine lysosomal processing of βAPPsw-paGFP, we treated cells with chloroquine and L685,458. After treatment with chloroquine, there was rapid trafficking of βAPPsw-paGFP signal to the lysosome where it accumulated in the photoactivation phase and then gradually decreased in brightness in the chase phase (Figure 6; Additional file 10: Video S7). This result was also seen after treatment with the γ-secretase inhibitor L685, 458 (Figure 6b; Additional file 11: Video S8). The loss of fluorescence signal in the chase phase as linear for both of these treatments was not significantly different from GFP photobleaching (Figure 6c and 6d). Together, these data suggests that the Swedish mutation accelerates the cleavage of APP at the lysosomal membrane, but does not have an effect on APP trafficking to the lysosome.

### APP interacts with adaptor protein AP-3

AP-3 is an adaptor protein that is integral to the direct delivery of lysosomal membrane proteins (LMPs) from the Golgi apparatus [66]–[70]. First, we sought to determine whether APP and AP-3 colocalize in neurons. Cortical neurons dissected from E15 mice were immunostained (DIV7) for the APP C-terminal and AP-3δ subunit, demonstrating colocalization. When analyzed by Imaris, 41.74 ± 2.60% (Mean ± SEM) of APP fluorescence was colocalized with AP-3 signal. We then depleted the δ subunit of AP-3 in SN56 cells using siRNA, as this has been shown to cause instability and degradation of the entire AP-3 complex [71]. We found that cells transfected with this siRNA expressed only 22.01 ± 11.98% (Mean ± SD) of the AP-3 δ subunit on Western blots (p < 0.05), while cells transfected with a control siRNA showed no significant change in AP-3 δ expression (Additional file 12: Figure S4a and b). We also observed this effect by immunostaining; cells transfected with siRNA (visualized by the Alexa fluor 647 label on the 5′ end of the control oligonucleotide) showed a marked decrease in AP-3δ staining while untransfected cells, or cells transfected with fluorescently labelled negative control siRNA only were unaffected (Additional file 12: Figure S4c).

To determine whether APP and AP-3 interact using the *in situ* proximity ligation assay (iPLA) which allows the study of low affinity interactions in-situ and has comparable accuracy to co-immunoprecipitation [72],[73]. Briefly, iPLA employs species-specific secondary antibodies bearing complementary DNA strands. If the two antibodies are within 40 nm, the DNA strands will hybridize, and the resulting sequence can be replicated, amplified, and labelled with fluorescent oligonucleotides. Pairs of interacting proteins were detected as red fluorescent dots/μm^3^.

SN56 cells were transfected with βAPP-CFP, and iPLA was used to determine the proximity of APP and AP-3. Cells mock transfected or transfected with negative control siRNA both demonstrate an interaction between APP and AP-3. Conversely, cells transfected with siRNA against AP-3 δ showed a marked decrease (approximately 98%) in fluorescent puncta signifying decreased interaction (Figure 6b and c).

### AP-3 Knockdown Disrupts Trafficking of APP to Lysosomes

**Figure 7:**
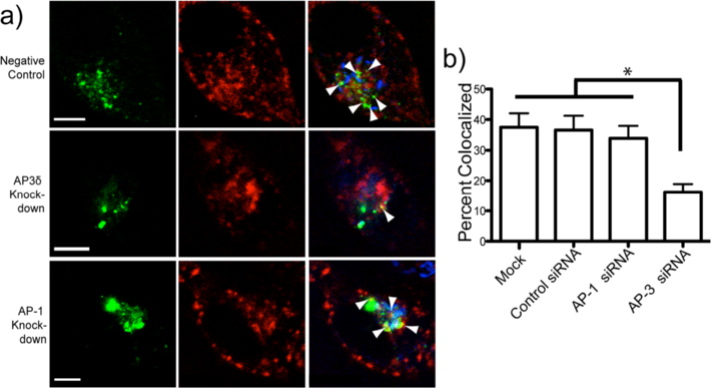
**AP-3 mediates direct trafficking of APP to lysosomes. a)** SN56 cells were transfected with βAPP-paGFP, LAMP1-mRFP, GalT-CFP, and either control siRNA, siRNA against AP-3δ mRNA or siRNA against AP-1γ. Cells were alternately photoactivated with 405 nm light and imaged in the Golgi for 15 minutes (scale bar represents 5 μm). White arrowheads in the merged image (far right panel) denote colocalized pixels. Scale bars represent 5 μm. **b)** Percent of βAPP-paGFP colocalizing with LAMP1-mRFP at the end of the 15-minute photoactivation period. (* = p < 0.05; Error bars represent standard deviation).

We hypothesized that we could disrupt the trafficking of APP to lysosomes by siRNA mediated knockdown of AP-3. As a control, we examined the effect of knockdown AP-1 (both AP-1a and AP-1b isoforms), which mediate cell surface trafficking, and trafficking to the basolateral membrane in epithelial cells [74],[75] and was effectively knocked down by siRNA (Additional file 4: Figure S3). As before, we co-transfected cells with βAPP-paGFP, GalT-CFP and LAMP1-mRFP with the addition of siRNA against either AP-1γ, AP-3δ, or a control siRNA. In cells transfected with active siRNA, a small amount of fluorescently tagged negative control siRNA was included as a marker to identify transfected cells. Cells were photoactivated in irradiation targets placed over the Golgi apparatus, and transport of APP was imaged over a 15-minute period and then analyzed for colocalization of photoactivated APP and LAMP1-mRFP. We found that cells transfected with control siRNA alone did not change βAPP-paGFP trafficking to lysosomes, as compared to cells not transfected with siRNA [37.47 ± 4.58% vs 36.57 ± 4.69%]. However, the siRNA against δ3 reduced APP transit to the lysosome to 16.24 ± 2.65% after 15 minutes of photoactivation. AP-1γ KD did not change the trafficking of APP from the TGN to lysosomes (33.86 ± 4.09%; Mean ± SEM) (Figure 7a and b). Therefore, AP-3 mediates rapid transport of APP to the lysosome, while AP-1 is not involved in the direct trafficking of APP to lysosomes, at least on the timescale examined here.

**Figure 8:**
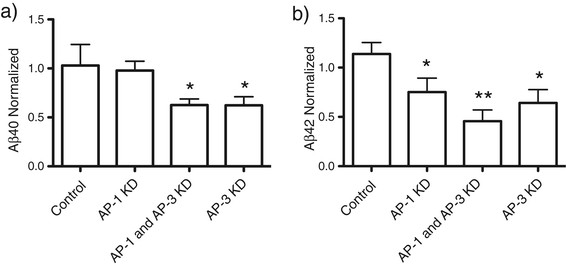
**AP-3 mediates processing to Aβ.** SN56 cells were transfected with βAPP-paGFP, LAMP1-mRFP, GalT-CFP, and either control siRNA, siRNA against AP-3δ mRNA or siRNA against AP-1γ. **a)** SN56 cells were co-transfected a plasmid expressing βAPPswe-CFP and with control siRNAs, siRNA against AP-1γ, siRNA against AP-1γ and AP-3δ combined, or siRNA against AP-3δ. Conditioned media was analyzed for **a)** Aβ40 or **b)** Aβ42 by ELISA. Experiments were performed 4 times, with each experiment consisting of 2 replicates. (* indicates significantly different from control p < 0.05; ** indicates significantly different from control and either AP-1 or AP-3 alone p < 0.05) Error bars represent SEM.

To determine the effect of AP-1 KD and AP-3 KD on APP processing, SN56 cells were transfected with βAPPsw-CFP and with negative control siRNA, AP-1 siRNA, AP-3 siRNA or a combination of AP-1 and AP-3 siRNAs. Two days after differentiation, culture media was taken from the cells and analyzed for Aβ40 and Aβ42 by ELISA (Invitrogen) (Figure 8a). AP-1 siRNA did not significantly alter the levels of Aβ 40 [106.0% ± 6.7% (mean ± SEM)], as compared to control. AP-3 siRNA and combined AP-3 and AP-1 siRNAs reduced the levels of Aβ40 53.9 ± 3.3% and 62.6 ± 6.2%, respectively (p < 0.5). For Aβ42 (Figure 8d), the control siRNA raised Aβ42 production slightly to 113.8 ± 121.6% (not significant) while siRNAs against AP-1 reduced Aβ42 levels in the media to 75.1 + 14.1% (not significant). Inhibitory siRNA to AP-3 alone or AP-1 and AP-3 together reduced the levels of Aβ42 to 64.2 ± 13.4 and 45.74 ± 11.4% respectively (P < 0.5) (Figure 8b). Therefore, AP-3 KD reduced the levels of Aβ40 and 42 in the media, and this effect was increased when AP-1 was knocked down as well.

## Discussion

In this work, we demonstrate the use of paGFP to study the intracellular trafficking of and clearance of APP. While paGFP has been used before to examine APP trafficking [28],[40],[76], this is the first report to follow APP from the Golgi apparatus into identified downstream compartments and examine its clearance pharmacologically. Although we had expected APP to traffic to the lysosome primarily via the plasma membrane, instead we observed rapid transport of APP to a LAMP1 compartment within seconds, similar to the behavior of LAMP1 [30]. Furthermore, we were able to follow the clearance of βAPP-paGFP in from lysosomes, essentially performing a pulse-chase experiment in this organelle in single cells. We show that βAPP-paGFP is cleared from the lysosome with first order kinetics, which is faster than the linear loss of fluorescence observed during photobleaching. The clearance of APP from lysosomes is sensitive to both chloroquine, a nonspecific inhibitor of lysosomal function, and L684, 458, a specific γ-secretase inhibitor. The direct trafficking of APP to this LAMP1 positive compartment is decreased by AP-3 knockdown. The depletion of AP-3 or AP-3 and AP-1 together results in a substantial reduction of Aβ in the media.

Because intracellular compartment markers localizations are not absolute, lysosomal markers can be found in endosomal compartments and vice versa [77],[78]. However, several features suggest that APP is moving to a *bona fide* lysosomal compartment. APP appears to be moving primarily to LAMP1 positive compartments, with less moving to compartments labeled with Rab5 and Rab9 (early and late) labeled compartments, suggesting that the main compartment receiving APP is LAMP1 positive and negative for rab5 and rab9. Furthermore, after the inhibition of degradation of APP with chloroquine or L685, 458, APP moves to LAMP1-labeled compartments and accumulates in these compartments, implying that they are terminal compartments of the endosomal lysosomal system. Furthermore, that AP-3 knockdown reduces the production and secretion of Aβ42 by 36% and Aβ40 by 47% suggests that the lysosome is a major site of APP processing and Aβproduction. These features suggest that APP is delivered predominantly to lysosomes.

Prior to this study, most APP trafficking studies suggested that APP primarily moved to the lysosome via the cell surface [79], however the evidence presented here suggests APP can traffic intracellularly from the Golgi to lysosomes. Kuentzel *et al.* found that less than ~20% of nascent APP is transported to the plasma membrane [80], suggesting that this direct lysosomal pathway maybe a major processing pathway of APP. LMPs are known to traffic to the lysosome by at least 2 different pathways. Some LMPs, like lysosomal acid phosphatase, transit first to the cell surface, and then cycle between the plasma membrane and endosomes repetitively before transport to the lysosome [81]. Other proteins, such as LAMP-1, transit intracellularly from the Golgi apparatus to lysosomes, without appearing at the cell surface [82],[83]. Our work is in agreement with of Castor *et al.*, who also demonstrated using a temperature-block paradigm that APP in the Golgi apparatus rapidly appears in a LAMP1-positive compartment rapidly after being released from the Golgi [84]. APP now has at least 4 distinct pathways to the lysosome – one from the cell surface through endocytosis into endosomes [85], one directly to lysosomes from the cell surface [50], one through autophagosomes [86], and finally the direct transport from the Golgi apparatus demonstrated here. This suggests that APP is a normal resident Lysosomal Membrane Protein.

This work is not intended to minimize the importance of APP’s cell surface trafficking. Indeed, AP-1 knockdown, which is predicted to interfere with APP trafficking to the cell surface, is able to substantially reduce Aβ production indicates that cell surface APP is still a significant source of Aβ. APP transiting to the cell surface is likely not being well visualized in this study for a number of reasons including: 1) activated APP-paGFP arriving at the membrane is diluted by a large amount of non-fluorescent APP already at the membrane, 2) APP has a relatively short half life at the membrane, estimated at 10 minutes [21],[87] and 3) we are imaging confocal sections and therefore seeing only a small fraction of the cell membrane itself. Indeed, Golgi apparatus-photoactivated βAPP-paGFP becomes visible at the plasma membrane when internalization is inhibited with a γ-secretase inhibitor [60],[61], demonstrating that APP can transit rapidly to the cell surface [80].

Several studies have suggested the presence of γ-cleavage in the lysosome. Our own studies have demonstrated that γ-secretase proteins and activity are present in highly purified lysosomes. Although many studies have observed γ-secretase function at neutral pH, we have found that γ-secretase activity within isolated lysosomal membranes possesses an acidic optimal pH [13]. Our findings are in agreement with previous findings that show a marked accumulation of APP in lysosomes after disruption of lysosomal pH, and inhibit the production of Aβ [17]. Furthermore, many other studies have documented the accumulation of APP fragments in lysosomes after inhibition of lysosomal enzymes or inactivation of PS1 [16],[18],[20],[88], suggesting a critical role for lysosomes in γ-secretase cleavage of APP. However, this is the first study to visualize accumulation of APP CTFs at the lysosomal membrane using a highly selective γ-secretase inhibitor [89]–[91].

Some investigators have suggested that APP (particularly APPsw) undergoes cleavage in the Golgi apparatus and in post-Golgi vesicles [62],[92]. Our data appears to show APP and APPsw accumulating stably in the Golgi apparatus when trafficking is blocked pharmacologically. Although it is impossible to rule out some processing of APP in the Golgi apparatus with these experiments, these data suggest that the Golgi apparatus is not a major site of Aβ production.

It is interesting that chloroquine and L684, 458 produce similar results. To our knowledge, chloroquine has never been shown to specifically affect the γ-secretase directly in cell free assays. Although our own data has suggests that γ-secretase functions at an acidic pH in the lysosome [13], most authors use a neutral pH [93] or mildly acidic pH [94] for this enzyme. However, efficient γ - secretase function requires the removal of the luminal domain of APP by β-cleavage [95]. The β-secretase is known to transit to the late endosomal/lysosomal compartments [96],[97]. β-cleavage may also be performed by lysosomal cathepsins [98],[99]. With a pH optimum below 4.5 [5],[100], β-secretase (like cathepsins) would function optimally in the lysosome. Therefore, the accumulation of APP in the lysosome after chloroquine could be by non-specifically inhibiting a luminal pH dependent β-secretase or β-like cleaving enzyme, which then secondarily inhibits γ-cleavage. When β-cleavage is accelerated by the APPsw mutation, APP-paGFP is cleared so rapidly that it cannot be imaged in the lysosome. This suggests that, rather than being a tightly controlled regulatory enzyme, the γ-secretase behaves more like a “proteasome of the membrane” whose job is to remove transmembrane stubs of proteins from the membrane [101].

Our results also demonstrate that lysosomal trafficking of APP may be an important mechanism of regulating APP cleavage. Specifically AP-3 knockdown reduces lysosomal trafficking of APP from the Golgi apparatus and reduces Aβ production and this effect is additive to AP-1 knock down. A wide range of other proteins likely also likely regulate APP processing by altering its trafficking including GGA1 [102],[103], X11a, Fe65 [104],[105], AP-4, [106], VPS35 [25] and SorLa [26]–[28]. These studies suggest that control of APP processing by intracellular trafficking may be crucial for regulating Aβ production.

## Conclusion

These findings may have broad importance for the pathophysiology of AD. This is because the lysosome’s biochemical milieu and acidic pH make it the ideal environment for the nucleation of amyloid fibrils [107],[108]. In fact, the lysosome has been proposed to be a site of Aβ aggregate seeding [109]–[111]. This development of Aβ aggregates has been shown to disrupt synapses [112] and membranes [113], and can lead to lysosomal rupture leading to cell death [114],[115]. Aβ may be secreted in exosomes, which are intraluminal vesicles released from the endosomal/ lysosomal system [57],[116]. Therefore, Aβ and its higher-order aggregates may be produced, nucleated, and secreted from lysosomes. The lysosome sits at a crossroad, as a site for the production and degradation of Aβ, as well as its fibrilogenesis. This work points to the importance of the lysosomal system in APP processing and its regulation in developing therapeutic treatments for AD.

## Materials and methods

### Antibodies

Antibodies used were: Rabbit anti-APP C-terminal (1:1000, Cat. No. A8717; Sigma), mouse anti-HA (1:1000, Cat. No. 12CA5;Roche); AP-3 δ3 subunit- mouse- SA4 (1:1000; Developmental Studies Hybridoma Bank); mouse anti-γ-adaptin (Cat No. 610386; BD Bioscience). Secondary antibodies used were donkey anti-mouse HRP (1: 10 000, Cat No. 711–0350150, Jackson Immunoresearch) and goat-anti rabbit HRP (1: 10 000; Biorad). α-tubulin was stained using a mouse monoclonal antibody (Cat No. T5168, Sigma). For immunostaining, donkey anti-rabbit Alexa Fluor 488 (A-11034; Invitrogen) and goat anti-mouse Alex Fluor 546 (A-11003; Invitrogen).

### Cell culture and transfection

SN56 (a gift from Dr. Jane Rylett) were grown in Dulbecco's Modified Eagle medium (DMEM) (Gibco) supplemented with 10%v/v of fetal bovine serum (FBS; Gibco) and 50 μg/ml of penicillin/streptomycin (P/S), in 5% CO_2_ at 37°C. Cells were split every 3 days. For confocal studies, 5 × 10^5^ cells were seeded on glass-bottomed culture dishes (MatTek) the day before transfection in DMEM supplemented with 10% FBS. Cells were transiently transfected using Lipofectamine (Invitrogen) according to manufacturer’s instructions. To differentiate the cells, the media was replaced 24 hours after transfection with DMEM supplemented with 50 μg/ml P/S and 1 mM dibutyryl cyclic AMP (dbcAMP; Sigma). Cells were differentiated for 24 hours and imaged or harvested. Primary cortical neurons were prepared from embryonic day 15 CD1 mouse embryos as described previously [117].

For silencing RNA (siRNA) mediated knockdown of the δ subunit of AP-3, Stealth Select 3 RNAi™ set (Invitrogen) was ordered. Sequence 3 of this set was found to knockdown the δ3 subunit (5′GAGAAGCUGCCUGUCCAGAAACAUA3′). The ubiquituously expressed γ1 subunit of AP-1 was knocked down using 5′UAAUAUAUCAUUCAUAGCU3′ with a 3′ TT overhang. Stealth RNAi™ siRNA Negative Control Med GC (12935–300; Invitrogen) was used as a control. The control RNAi was tagged on the 5′ end with Alexa 647 to determine which cells were transfected with siRNA. For each 35 mm dish, 200nM of siRNA was transfected using Lipofectamine 2000 according to manufacturer’s instructions. During the knockdown experiments, 1nM of negative control siRNA was co transfected with the siRNA against δ3 or γ1 to confirm the transfection of siRNA into the cell.

### DNA Constructs

A cDNA encoding APP 750- yellow fluorescent protein (YFP) was a kind gift from Dr. Bradley Hyman (Massachusetts General Hospital). Constructs expressing full length or shortened (last 112 amino acids) APP (βAPP) with an amino terminal hemagglutination (HA) tag and enhanced cyan FP (eCFP) on the carboxyl terminus were generated as previously described [50]. Plasmids expressing photoactivatable GFP (paGFP) was a kind gift of Dr. Jennifer Lippincott-Schwartz [30]. βAPP was recloned such that paGFP is placed on the C-terminal cytoplasmic tail of the protein. The Swedish mutation was introduced into the βAPP-ECFP construct using PCR [50], and was recloned into the paGFP vector.

Rab5-mRFP, Rab9-mCherry, and LAMP1-mRFP were generated as previously described [50]. VSVG-paGFP construct was purchased from Addgene (http://www.addgene.org).

### Confocal microscopy

A Zeiss LSM-510 META laser- scanning microscope using a Zeiss 63× 1.4 numerical aperture oil immersion lens was used to take images (Carl Zeiss, Oberkochen, Germany). The optical section thickness was typically 1 μM. To visualize Alexa Fluor 488 and paGFP fluorescence, they were excited with a 488 nm laser and filtered using a band pass (BP) 500-530-nm emission filter set. For Alexa Fluor 546, mCherry and mRFP fluorescence, a 543 nm excitation laser and BP 560–615 filter set was used. To collect ECFP fluorescence, a BP 475–525 emission filter set was used after excitation with a 458 nm lasers. Alexa Fluor 647 fluorescence was imaged using 633 nm excitation laser, and a LP 650 filter.

### Live cell imaging

For live cell imaging, the cells were washed twice with PBS, and the culture media was replaced with 37°C Hank’s Balanced Salt Solution (HBSS; Cat. No. 14025–092, Invitrogen). To maintain a constant temperature of 37°C, the 35 mm plate was placed on a heated stage (heated insert P; PeCon GmbH) connected to a Tempcontrol 37–2 digital 2-channel (PeCon GmbH).

Using the Ziess Physiology package, regions of interest (ROI) were selected in the Golgi apparatus, which was demarcated by GalT-CFP fluorescence and these were carefully monitored during the experiment to ensure that they remained over the Golgi apparatus if the cell or the Golgi apparatus apparatus moved. In a typical experiment, cells were imaged approximately every 30 seconds. For the first 15 minutes, ROIs in the Golgi apparatus were irradiated with 405 nm laser light to photoactivate APP-paGFP before imaging. After the initial 15-minute pulse period, images were take without irradiation and the movement/degradation of paGFP fluorescence was followed for approximately 45 minutes.

To inhibit APP-paGFP cleavage, cells were treated with cholorquine (Cat. No. C6628, Sigma) or L685, 458 (Cat. No. 565771, EMD Millipore). Cells were treated with 100 μM choloroquine 30 minutes before imaging to deacidify lysosomes. Deacidification of lysosomes was confirmed using 75nM Lysosensor™ Green (Cat. No. L-7534, Invitrogen). To inhibit cleavage using a specific γ-secretase inhibitor, SN56 cells were treated with 0.5 μM L685, 458 for 24 h before imaging.

### Colocalization analysis

Colocalization analysis was performed on using Imaris 7.0 Imaris Colocalization module (Biplane). Imaris software was used to create IsoSurfaces corresponding to the paGFP and RFP fluorescence channels following the manufacturer’s directions (www.bitplane.com) [118]. This is a computer assisted method to set fluorescence intensity thresholds to detect fluorescence in an organellar distribution that can then be used to automatically follow fluorescence intensity and colocalization over time. The co-localization of APP and LAMP1 over time was plotted using Prism 5.0 software (Graphpad, La Jolla CA) and curves were fit using the nonlinear regression by least squares to fit a one phase exponential decay.

To colocalize AP-3δ and APP we adopted a strategy we have previously employed [50] and described by Hutcheon *et al.*[119] (also discussed in [120],[121]), which sets thresholds based on a fixed percentage of the brightest pixels in an image. This allows for the identification of positive pixels that is unbiased (it does not require the judgment of the observer on an image to image basis) and is relatively unaffected by parameters of image acquisition or the level of protein expression. To colocalize AP-3δ and APP, the brightest 2% of pixels was selected, and the percentage of pixels colocalized was recorded [50]. Prism Graphpad 5.0b was used for all graphing and statistical analysis. A One-way ANOVA was performed with a Dunn’s post-hoc test, and P values under 0.05 were considered significant.

### Immunostaining

SN56 cells or mouse cortical neurons were fixed for 15 minutes with 4% paraformaldehyde (Alfa Aesar; Cat No. 43368). Cells were permeabilized for 5 minutes with 0.1% TritonX-100 in PBS and blocked with 2% BSA for 1 h. Cells were incubated with primary antibodies overnight at 4°C, washed twice with PBS, and stained with secondary antibody for 1 h. After staining, confocal plates were store at 4°C in PBS, and coverslips were mounted on glass slides with ImmunoMount (Fisher) and stored at 4°C.

### Proximity ligation assay (PLA)

SN56 cells were transiently transfected with βAPP-eCFP, and fixed for 15 minutes with 4% paraformaldehyde. Cells were permeabilized and blocked in the same manner as immunostaining. PLA was performed using a commercially available kit (Duolink; Olink Bioscience) according to manufacturer’s instructions. Briefly, primary antibodies were washed off cells with PBS, and species specific PLA secondary probes were applied to cells. If secondary PLA probes are within 40 nm of each other, their complementary DNA strands are ligated and are amplified. Complementary fluorescent oligonucleotides bind to the amplified sequence, which results in a fluorescent dot where there are two interacting proteins.

### Cell Lysis and Western Blots

SN56 cells 1.5×10^6^ cells were seeded on 60 mm tissue culture dishes (Becton Dickinson) and transfected with plasmids or siRNA using Lipofectamine 2000 according to manufacturer’s instructions. Cells were harvested in lysis buffer (1% Nonidet P-40, 150 mm NaCl, 50 mM Tris-Cl) supplemented with pepstatin and complete protease inhibitor cocktail (Roche). Lysates were clarified by centrifugation at 13,000 g for 20 min. To facilitate equal loading, the total amount of total protein was determined by bicinchoninic acid (BCA; Thermo Fisher Scientific). Samples were electrophoresed on SDS-PAGE and transferred to PVDF membrane (Cat No. 162–01777; Biorad). Densitometry was performed in ImageJ (NIH), and was normalized to α-tubulin band density. Graphs were plotted in Prism 5.0b (Graphpad, La Jolla, CA), a one way ANOVA was performed with a Tukey’s post-hoc test. Results were significant if p < 0.05.

## Abbreviations

AD: Alzheimer’s disease

APP: Amyloid precursor protein

Aβ: Beta amyloid

AP-1: Adaptor protein-1

AP-3: Adaptor protein 3

GalT: Glactosyltransferase

GFP: Green fluorescent protein

paGFP: Photoactivatable GFP

VSVG: Vesicular stomatitis virus glycoprotein

## Competing interest

The authors have no competing interests.

## Authors’ contributions

The JT was responsible for designing and carrying out most of the experiments in this manuscript. CS established protocols for neuronal culture and PLA. SHP conceived of the study, and participated in its design and coordination and helped to draft the manuscript. All authors read and approved the final manuscript.

## Additional files

## Supplementary Material

Additional file 1: Figure S1. βAPP-paGFP and full-length APP are cleaved by that γ-secretase in a similar manner. SN56 cells were transiently transfected with plasmids expressing GFP, full-length APPpaGFP (FL-APP-paGFP), or βAPP-paGFP. Twenty-four hours before harvesting protein for western blotting, cells were treated with DMSO or with L685, 458. Cell lysate was run on a 12% SDS polyacrylamide gel, and transferred onto nitrocelluose membrane. Membrane was probed for APP using APP C-terminal antibody (Sigma). Membranes were reprobed for α-tubulin, as a loading control. Full length APP-paGFP is cleaved to produce fragments of the predicted size, with a b-cleaved fragment at ~37 kDA (which is GFP + the 10 kDa b-cleaved APP). The addition of the γ-secretase inhibitor L685, 458 causes the accumulation of the 37 kDa band). This pattern is repeated for the shorter βAPP-paGFP construct. Click here for file

Additional file 2: Figure S2. Most of the trafficked APP in the cell is uncleaved. SN56 cells were transiently transfected with plasmids expressing βAPP-CFP, and immunostained with an anti-HA antibody, which binds to the HA-epitope on the N-terminus of the construct. In the merged image, it is possible to see that there is extensive colocalizaition of the N-terminal HA and the C-terminal CFP tag, implying that much of the intracellular APP is being trafficked uncleaved. Click here for file

Additional file 3: Video S1/Figure 1. APP is trafficked rapidly to the lysosome and cleared. SN56 cells were transiently transfected with GalT-CFP to identify the Golgi apparatus, LAMP1-mRFP to identify lysosomes, and βAPP-paGFP. Irradiation targets (circles) were drawn over the Golgi apparatus and the were irradiated with 405 nm laser light, alternating with imaging for 15 minutes (indicated by the green word ‘photoactivating’ on the images. Cells were then followed in a ‘chase period’ imaging every 30 seconds for the time indicated. Click here for file

Additional file 4: Figure S3. Colocalization of photo-activated APP-paGFP with LAMP1. SN56 cells were transiently transfected with plasmids expressing GFP, βAPP-paGFP and GalT-CFP. **a)** Shows the initial image of an SN56 cell before photoactivation, with the Golgi apparatus labelled blue (GalT-CFP) and lysosomes labelled red with LAMP1-mRFP. Thresholds were set in the red and blue channels to identify the Golgi apparatus and Lysosomes using Imaris software, and a colocalization channel is generated and overlaid in white. Although the Golgi apparatus and Lysosomes are closely apposed, the fluorescent protein markers demonstrate minimal colocalization. Panel **b** shows the same cell after 15 minutes of Golgi-targeted photoactivation with activated βAPP-paGFP in green and lysosomes labelled red with LAMP1-mRFP. The inset is magnified as figure **c**. Panel **c** shows the red LAMP1-mRFP and green photoactivated βAPP-paGFP channels separately. Thresholds were set in the red and green channels to identify the lysosomes and the majority of the APP fluorescent signal using Imaris software, and a colocalization channel is generated and overlaid in white. This channel demonstrates extensive colocalization of APP-paGFP and LAMP1. Furthermore, many regions of APP labelled fluorescence have the same shape as the underlying LAMP1 label, implying that they are indeed colocalized in these confocal images. Click here for file

Additional file 5: Video S2/Figure 1. APP paGFP is accurately photoactivated in the Golgi apparatus. SN56 cells were transiently transfected with GalT-CFP to identify the Golgi apparatus, LAMP1-mRFP to identify lysosomes, and βAPP-paGFP and were treated with Nocodozole to block exit from the Golgi. Irradiation targets (circles) were drawn over the Golgi apparatus and the were irradiated with 405 nm laser light, alternating with imaging for 15 minutes (indicated by the green word ‘photoactivating’ on the images. Cells were then followed in a ‘chase period’ imaging every 30 seconds for the time indicated. Photoactivated βAPP-paGFP can be seen accumulating in the Golgi. Click here for file

Additional file 6: Video S3/Figure 3. APP processing in the lysosome is blocked by Chloroquine in the lysosome. SN56 cells were transiently transfected with GalT-CFP to identify the Golgi apparatus, LAMP1-mRFP to identify lysosomes, and βAPP-paGFP. Cells were pretreated with 100 μM chloroquine 30 minutes before imaging. Irradiation targets (circles) were drawn over the Golgi apparatus and the were irradiated with 405 nm laser light, alternating with imaging for 15 minutes (indicated by the green word ‘photoactivating’ on the images. Cells were then followed in a ‘chase period’ imaging every 30 seconds for the time indicated. Photoactivated βAPP-paGFP can be seen accumulating in lysosomes. Click here for file

Additional file 7: Video S4/Figure 3. APP processing in the lysosome is blocked by L685, 458 in the lysosome. SN56 cells were transiently transfected with GalT-CFP to identify the Golgi apparatus, LAMP1-mRFP to identify lysosomes, and βAPP-paGFP. Cells were pretreated with 0.5 μM L685, 458 overnight. Irradiation targets (circles) were drawn over the Golgi apparatus and the were irradiated with 405 nm laser light, alternating with imaging for 15 minutes (indicated by the green word ‘photoactivating’ on the images. Cells were then followed in a ‘chase period’ imaging every 30 seconds for the time indicated. Photoactivated βAPP-paGFP can be seen accumulating in lysosomes. Click here for file

Additional file 8: Video S5/Figure 4. APPsw trafficking is rapidly processed. SN56 cells were transiently transfected with GalT-CFP to identify the Golgi apparatus, LAMP1-mRFP to identify lysosomes, and βAPPsw-paGFP. Irradiation targets (circles) were drawn over the Golgi apparatus and the were irradiated with 405 nm laser light, alternating with imaging for 15 minutes (indicated by the green word ‘photoactivating’ on the images. Cells were then followed in a ‘chase period’ imaging every 30 seconds for the time indicated. APPsw is cleaved so rapidly that it is unable to accumulate in any compartment. Click here for file

Additional file 9: Video S6/Figure 4. APPsw is not cleared in the Golgi apparatus. SN56 cells were transiently transfected with GalT-CFP to identify the Golgi apparatus, LAMP1-mRFP to identify lysosomes, and βAPPsw-paGFP and were treated with 66 μM nocodazole and 10 μM cytochalasin. Irradiation targets (circles) were drawn over the Golgi apparatus and the were irradiated with 405 nm laser light, alternating with imaging for 15 minutes (indicated by the green word ‘photoactivating’ on the images. Cells were then followed in a ‘chase period’ imaging every 30 seconds for the time indicated. Photoactivated βAPPsw-paGFP can be seen accumulating in the Golgi. Click here for file

Additional file 10: Video S7/Figure 5. APPSw processing in the lysosome is blocked by chloroquine. SN56 cells were transiently transfected with GalT-CFP to identify the Golgi apparatus, LAMP1-mRFP to identify lysosomes, and βAPPsw-paGFP and were treated with 100 μM chloroquine. Irradiation targets (circles) were drawn over the Golgi apparatus and the were irradiated with 405 nm laser light, alternating with imaging for 15 minutes (indicated by the green word ‘photoactivating’ on the images. Cells were then followed in a ‘chase period’ imaging every 30 seconds for the time indicated. Photoactivated βAPPsw-paGFP can be seen accumulating in lysosomes. Click here for file

Additional file 11: Video S8/Figure 4. APPSw processing in the lysosome is blocked by L685, 458; γ-cleavage occurs in the lysosome. SN56 cells were transiently transfected with GalT-CFP to identify the Golgi apparatus, LAMP1-mRFP to identify lysosomes, and βAPPsw-paGFP and were treated with 0.5 μM L685, 458 overnight. Irradiation targets (circles) were drawn over the Golgi apparatus and the were irradiated with 405 nm laser light, alternating with imaging for 15 minutes (indicated by the green word ‘photoactivating’ on the images. Cells were then followed in a ‘chase period’ imaging every 30 seconds for the time indicated. Photoactivated βAPPsw-paGFP can be seen accumulating in lysosomes. (MOV 449 kb) Click here for file

Additional file 12: Figure S4. Knockdown of AP3 and AP1 by siRNA. **a)** SN56 cells were transfected with fluorescently-tagged control siRNA or AP-3δ and fluorescently tagged siRNA. Western blot demonstrating that AP-3δ siRNA decreases AP-3δ protein. Blots were stripped and re-probed with anti-tubulin antibody as a loading control. **b)** Western blots (from a) were scanned and analyzed using densitometry (ImageJ) and graphed. Error bars represents standard error of the mean. (* = p < 0.05). **c)** SN56 cells were transfected with fluorescently tagged control siRNA or anti-AP-3δ and fluorescently tagged siRNA (purple). Cells were the immunostained to detect AP-3δ (red)**.** Fluorescent images overlayed with white light images to delimit the cell body. (scale bars represents 5 μm). **d)** SN56 cells were transfected with control siRNA or siRNA against AP-1γ. Western blot demonstrating that AP-1γ siRNA decreases AP-1 protein. Blots were stripped and re-probed with anti-tubulin antibody as a loading control. **e)** Western blots (from d) were scanned and analyzed using densitometry (ImageJ) and graphed. Error bars represents standard error of the mean. (* = p < 0.05). Click here for file
