# Supplementary figures and images for: The Amyloid Precursor Protein is rapidly transported from the Golgi apparatus to the lysosome and where it is processed into beta-amyloid
Source: Mol Brain. 2014 Aug 1;7:54. doi: 10.1186/s13041-014-0054-1 (PMC4237969; doi:10.1186/s13041-014-0054-1)

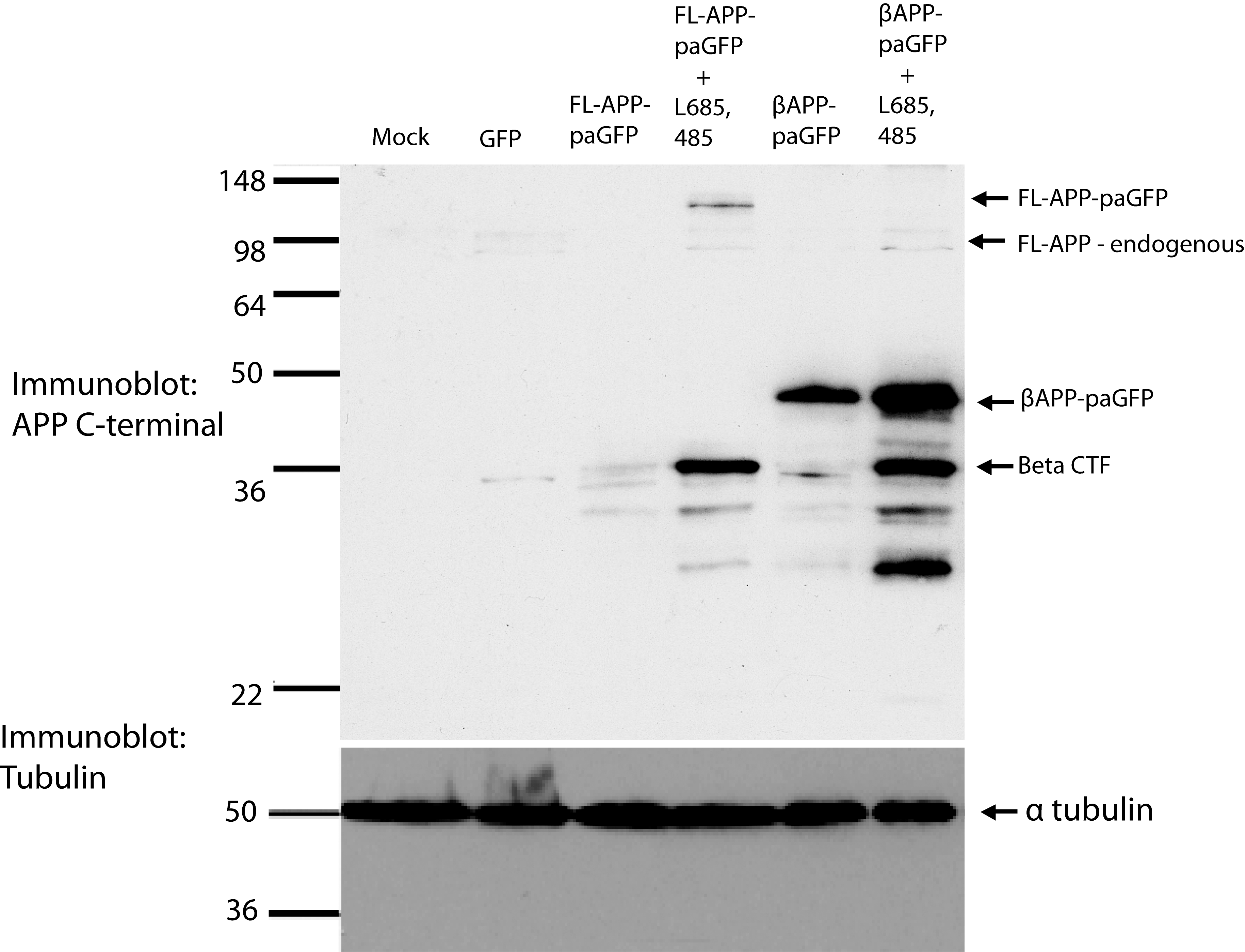

Supplement: Additional file 1: Figure S1. — βAPP-paGFP and full-length APP are cleaved by that γ-secretase in a similar manner. SN56 cells were transiently transfected with plasmids expressing GFP, full-length APPpaGFP (FL-APP-paGFP), or βAPP-paGFP. Twenty-four hours before harvesting protein for western blotting, cells were treated with DMSO or with L685, 458. Cell lysate was run on a 12% SDS polyacrylamide gel, and transferred onto nitrocelluose membrane. Membrane was probed for APP using APP C-terminal antibody (Sigma). Membranes were reprobed for α-tubulin, as a loading control. Full length APP-paGFP is cleaved to produce fragments of the predicted size, with a b-cleaved fragment at ~37 kDA (which is GFP + the 10 kDa b-cleaved APP). The addition of the γ-secretase inhibitor L685, 458 causes the accumulation of the 37 kDa band). This pattern is repeated for the shorter βAPP-paGFP construct. [file s13041-014-0054-1-S1.tiff]

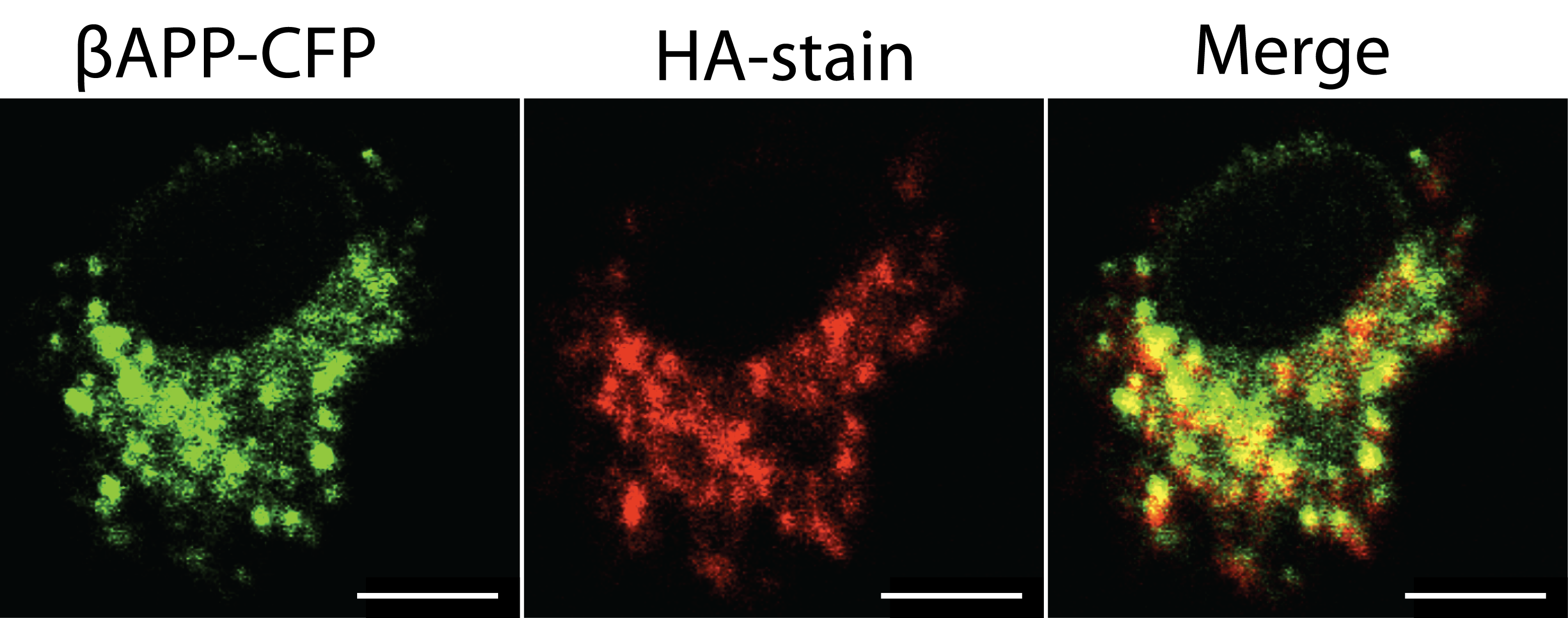

Supplement: Additional file 2: Figure S2. — Most of the trafficked APP in the cell is uncleaved. SN56 cells were transiently transfected with plasmids expressing βAPP-CFP, and immunostained with an anti-HA antibody, which binds to the HA-epitope on the N-terminus of the construct. In the merged image, it is possible to see that there is extensive colocalizaition of the N-terminal HA and the C-terminal CFP tag, implying that much of the intracellular APP is being trafficked uncleaved. [file s13041-014-0054-1-S2.tiff]

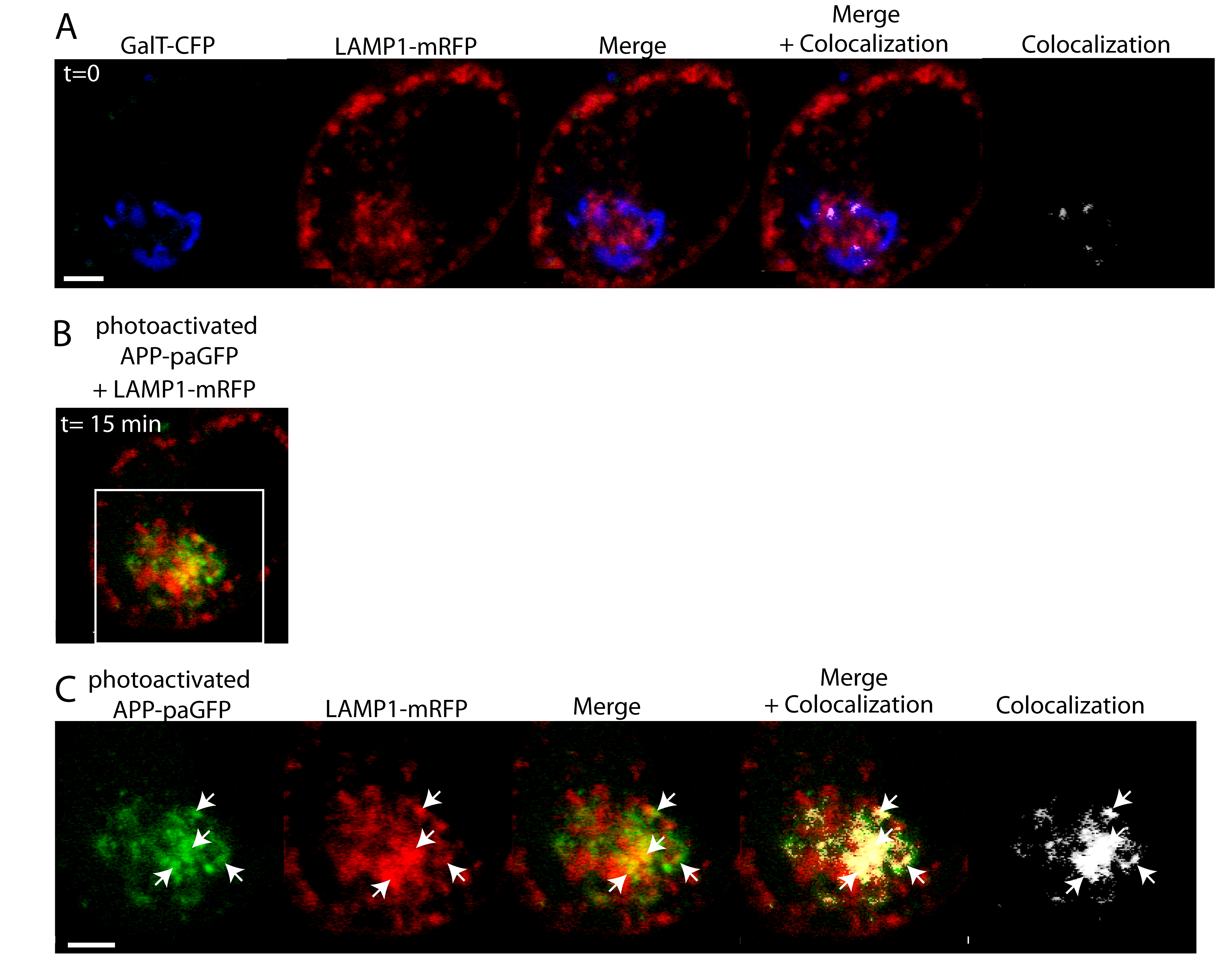

Supplement: Additional file 4: Figure S3. — Colocalization of photo-activated APP-paGFP with LAMP1. SN56 cells were transiently transfected with plasmids expressing GFP, βAPP-paGFP and GalT-CFP. a) Shows the initial image of an SN56 cell before photoactivation, with the Golgi apparatus labelled blue (GalT-CFP) and lysosomes labelled red with LAMP1-mRFP. Thresholds were set in the red and blue channels to identify the Golgi apparatus and Lysosomes using Imaris software, and a colocalization channel is generated and overlaid in white. Although the Golgi apparatus and Lysosomes are closely apposed, the fluorescent protein markers demonstrate minimal colocalization. Panel b shows the same cell after 15 minutes of Golgi-targeted photoactivation with activated βAPP-paGFP in green and lysosomes labelled red with LAMP1-mRFP. The inset is magnified as figure c. Panel c shows the red LAMP1-mRFP and green photoactivated βAPP-paGFP channels separately. Thresholds were set in the red and green channels to identify the lysosomes and the majority of the APP fluorescent signal using Imaris software, and a colocalization channel is generated and overlaid in white. This channel demonstrates extensive colocalization of APP-paGFP and LAMP1. Furthermore, many regions of APP labelled fluorescence have the same shape as the underlying LAMP1 label, implying that they are indeed colocalized in these confocal images. [file s13041-014-0054-1-S4.tiff]

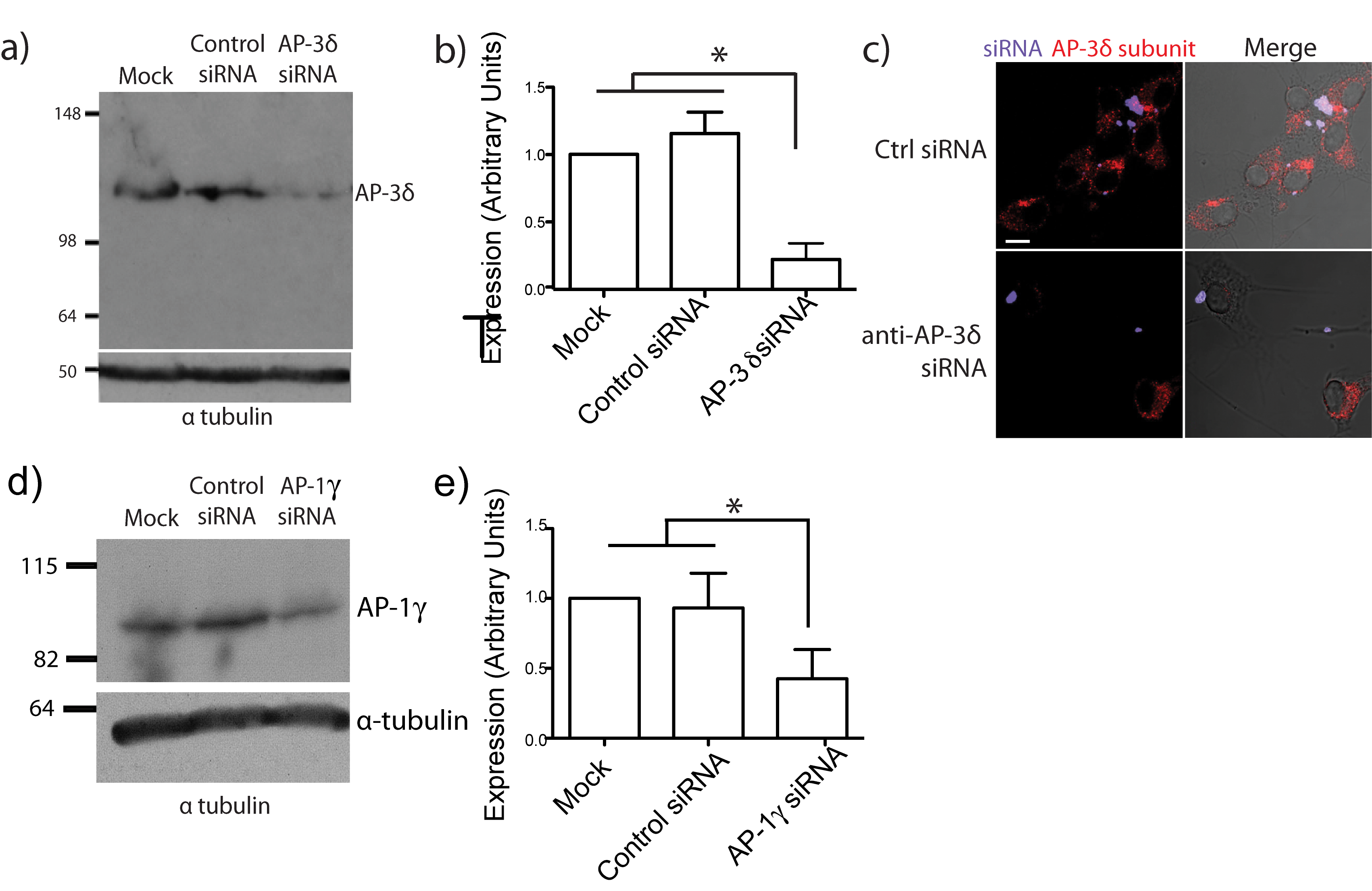

Supplement: Additional file 12: Figure S4. — Knockdown of AP3 and AP1 by siRNA. a) SN56 cells were transfected with fluorescently-tagged control siRNA or AP-3δ and fluorescently tagged siRNA. Western blot demonstrating that AP-3δ siRNA decreases AP-3δ protein. Blots were stripped and re-probed with anti-tubulin antibody as a loading control. b) Western blots (from a) were scanned and analyzed using densitometry (ImageJ) and graphed. Error bars represents standard error of the mean. (* = p < 0.05). c) SN56 cells were transfected with fluorescently tagged control siRNA or anti-AP-3δ and fluorescently tagged siRNA (purple). Cells were the immunostained to detect AP-3δ (red). Fluorescent images overlayed with white light images to delimit the cell body. (scale bars represents 5 μm). d) SN56 cells were transfected with control siRNA or siRNA against AP-1γ. Western blot demonstrating that AP-1γ siRNA decreases AP-1 protein. Blots were stripped and re-probed with anti-tubulin antibody as a loading control. e) Western blots (from d) were scanned and analyzed using densitometry (ImageJ) and graphed. Error bars represents standard error of the mean. (* = p < 0.05). [file s13041-014-0054-1-S12.tiff]
